# *CytoML* for cross‐platform cytometry data sharing

**DOI:** 10.1002/cyto.a.23663

**Published:** 2018-12-14

**Authors:** Greg Finak, Wenxin Jiang, Raphael Gottardo

**Affiliations:** ^1^ Program in Biostatistics Bioinformatics and Epidemiology, Vaccine and Infectious Disease Division Fred Hutchinson Cancer Research Center Seattle Western Australia

**Keywords:** standards, interoperability, data sharing, data analysis, bioinformatics, R/bioconductor

## Abstract

*CytoML* is an R/Bioconductor package that enables cross‐platform import, export, and sharing of gated cytometry data. It currently supports Cytobank, FlowJo, Diva, and R, allowing users to import gated cytometry data from commercial platforms into R. Once data are available in R, the data can be further manipulated. For example it can be combined with other computational and analytic approaches, and the results can be exported to FlowJo or Cytobank to be explored by researchers using those platforms. We demonstrate how *CytoML* and related R packages can be used as a tool to import, modify and export several samples stained with the T cell panel from the FlowCAP IV Lyoplate data set. Once imported, the gating is modified using computational approaches, and exported for visualization in Cytobank and FlowJo. We further show how *CytoML* can be used to import gated data from a publicly accessible mass cytometry experiment from Cytobank. *CytoML* is the only tool that allows such sharing of gated cytometry data between researchers working across different platforms, and it will serve as a useful tool for validating and verifying the reproducibility of analyses. © 2018 The Authors. Cytometry Part A published by Wiley Periodicals, Inc. on behalf of International Society for Advancement of Cytometry.

## Introduction

Reproducibility is critical for flow cytometry since it is a fundamental assay used for immune monitoring and to define endpoints for clinical trials and for diagnosis 1, 2, 3, 4. Reproducibility receives a lot of attention from experimentalists; the optimized multicolor immunophenotyping panels (OMIP) publications in the journal *Cytometry A* are an example of such an effort to publish validated staining panels, as are the Lyoplate and Euroflow studies which examined the influence of experimental factors and fully standardized pipelines on reproducibility and variability 5, 6, 7, 8, 9, 10. Computational analysis has been another major research effort to try and tackle reproducibility by eliminating the human element of analysis 7, 11, 12, 13, 14, 15. Data standards also play an important role by defining how data should be represented and annotated, they enable interoperability between instruments and analytic platforms 16, 17, 18, 19, 20, 21, 22.

Although flow and mass cytometry are increasing in dimensionality and throughput and despite the growing adoption of computational approaches for dimension reduction and analysis of these data, traditional bivariate gating has a solid foot hold and a well‐established place in the field due to its simplicity and ease of interpretation 23, 24.

Hierarchical gating is actually rather complex. It depends on many upstream decisions about how data are transformed, and implicitly conditions on upstream cell populations. In order to reproduce a hierarchical gating strategy exactly, this implicit and explicit information must be captured along with the gate boundaries.

The Gating‐ML standard was developed to tackle this problem 22, 25. It is designed to describe the gates that define different cell populations and the hierarchical relationships between them. It has been a critical contribution to the field, although it has not been widely adopted by software platforms and not always to the exact specification.

Despite the development of standards, tools that enable data sharing are still lacking. There is no software that allows gated and analyzed data to be exported from one platform and imported into another to reproduce an analysis from raw FCS files. This functionality is critical to allow verifiable reproducibility of experimental results and to develop the current state of the art of computational analysis. Concretely, manual analyses performed in FlowJo, Diva, or Cytobank should be accessible within R/Bioconductor 26 so that they can be compared with computational approaches, so that cell population statistics can be reliably extracted for statistical analysis and reporting, and so that new analytic approaches, not envisioned by the data creators, can be applied to the data. Likewise, computational analyses performed in R/Bioconductor should be accessible for exploration by researchers more comfortable working in other environments in order to provide critical assessment of results.

Here, we present a new R package, *CytoML*, that enables this type of cross‐platform data sharing. It reads the various flavors of Gating‐ML implemented by Diva, FlowJo, and Cytobank and imports these data analysis formats (named *workspaces* here) together with the raw FCS files, data transformations, and compensation matrices into R/Bioconductor in order to faithfully reproduce data analysis from these platforms. It leverages the *flowWorkspace* R package 27 to reconstruct the gated analysis in R, where it can be interrogated, explored, plotted, modified, and exported via *CytoML* to a FlowJo or Cytobank workspace. By implementing published data standards and the R/Bioconductor computational flow framework, *CytoML* implements an interface for exchanging gated cytometry data and reproducing analyses across different platforms.

We demonstrate how to use *CytoML* to import data from FlowJo, Diva, and Cytobank, visualize those analyses in R/Bioconductor using *ggcyto*, make modifications with *openCyto*, and then re‐export them for exploration in FlowJo and Cytobank 23, 28.

### Availability

*CytoML* is open‐source and available through Bioconductor (https://doi.org/doi:10.18129/B9.bioc.CytoML) and from the RGLab GitHub site (http://github.com/RGLab/CytoML). A Docker container with an installation of *RStudio*, *CytoML,* and other R flow cytometry tools is available from Docker Hub (https://hub.docker.com/r/gfinak/opencyto). A fully reproducible workflow of the data and results presented in this manuscript is available from the Supporting Information Material as well as on‐line at http://rglab.org/CytoML. The versions of *CytoML* and other packages required to reproduce these results are listed in that document.

### Implementation

Diva, FlowJo, and Cytobank store data transformations, compensation matrices, gates and their hierarchical relationships, sample meta‐data and other information required to reproduce a gating analysis in XML files termed “workspaces.” The XML used by each platform is a variant of Gating‐ML. *CytoML* implements a parser for the different flavors of Gating‐ML and maps the different analytic objects to the core cytometry data structures in R (*GatingSet* and *GatingHierarchy*). These are implemented in the *flowWorkspace* package and represent hierarchically gated FCM analyses in R/Bioconductor. The power of *CytoML* is the ability to reproduce these external analyses from raw cell level data. Each object carries with it all the necessary information, parsed by *CytoML* from the platform‐specific XML, to faithfully and completely reproduce the analysis in R.

### Compensation and Data Transformations

Compensation matrices and data transformations are chosen to faithfully reproduce an analysis from a workspace. *CytoML* detects when a custom compensation matrix is used for analysis (e.g., in FlowJo) and selects it automatically. The user is not free to change compensation or transformation parameters *for data import*, since this would alter the location of cells in gates and the imported analysis would no longer be a faithful reproduction of the original analysis.

*CytoML* converts imported data to a common representation that is shared by computational gating tools in R, allowing users to mix computational approaches with manual analysis 23, 29. Conversely, data in this common format (the *GatingHierarchy* or *GatingSet* representation implemented in the *flowWorkspace* package) can be exported to FlowJo or Cytobank compatible XML, allowing data analyzed in Bioconductor to be shared with investigators using those tools.

### Access to Cell Level Data

Once an analysis is imported into R, users have access to single‐cell resolution data, including the ids of individual cells and their memberships within different cell populations or gates. The *flowWorkspace* APIs getData() and getIndices() will return the set of cells belonging to a specific cell population or the indices of cells belonging to a specific population (see Supporting Information Material: “accessing cell level data”). We show how this can be used to compute the F measure comparing *FlowSOM* 30 clusters against the manual CD4+ T cell gate in the Supporting Information Material.

## Use Cases

We demonstrate import and export of manually and computationally gated data to and from FlowJo and Cytobank workspace formats. Diva import is also supported via the openDiva(), parseWorkspace() *CytoML* interfaces (APIs) (see Supporting Information Material: “Importing Diva XML”). A reproducible example of the workflow presented in this article is available at http://rglab.org/CytoML/ as well as in the Supporting Information Material for this manuscript. An overview of the CytoML workflow is presented in Supporting Information Figure S1.

### Data Sets

We demonstrate the use of *CytoML* on two data sets. The first is Lyoplate data from a FlowCAP IV study 7, where triplicate Cytotrol control cell samples were distributed to nine centers for staining, analysis, and gating using a variety of Lyoplate panels. Data were gated in three ways: manually at each center by different analysts, manually by a single analyst, and using computational methods, then the contribution of different factors, namely the gating method and the staining, to overall variability was assessed. The complete data are available from ImmuneSpace (https://immunespace.org/\_webdav/HIPC/Lyoplate/\%40files//gated\_data/pop\_renamed/manual‐gslist‐tcell.tar.gz) (free ImmuneSpace sign up and login required) 31, 32. The FCS files used here for demonstration are distributed with the *flowWorkspaceData* package 33 in Bioconductor.

The second data set is a public Cytobank experiment, “Kinase Inhibitor‐Treated Reprogramming MEFs” 34. This was a study that used mass cytometry to analyze cellular reprogramming by measuring markers of pluripotency, differentiation, cell‐cycle status, and cellular signaling and performed time‐resolved progression analysis of the resulting data. The URL for this study is (https://community.cytobank.org/cytobank/experiments/43281), with Cytobank ID 43281.

### Importing Gated Data from FlowJo

**Figure 1:**
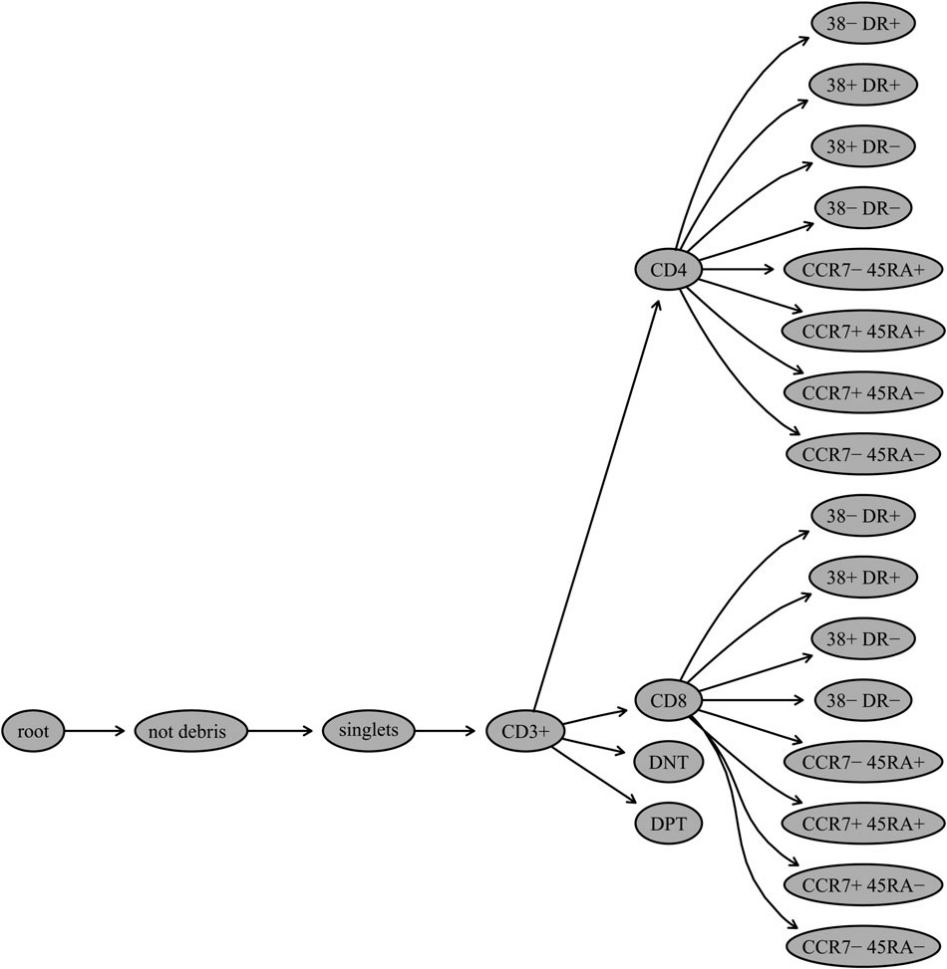
The manual gating tree for the Cytotrol T cell panel from the FlowCAP IV Lyoplate data set.

**Figure 2:**
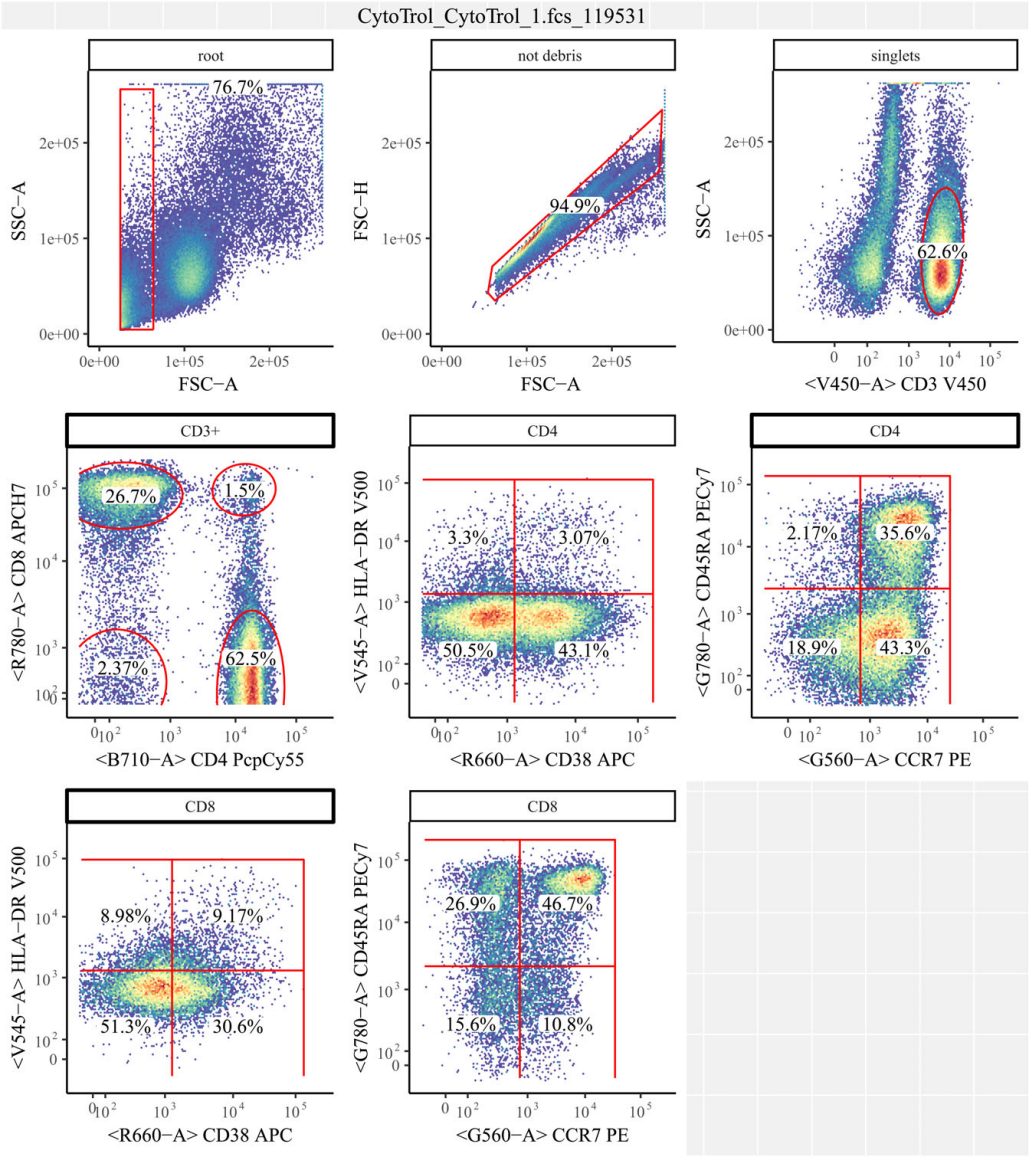
A *ggcyto* visualization of the manual gating scheme for one sample of the Cytotrol T cell data from the Lyoplate FlowCAP IV data, imported into R/Bioconductor using *CytoML* and flowWorkspace. [Color figure can be viewed at wileyonlinelibrary.com].

FlowJo import is demonstrated on a pair of Cytotrol samples stained using the Lyoplate T‐cell panel. The FlowJo manual gates are imported using the openWorkspace() and parseWorkspace() APIs, creating a *GatingSet* object in R. After import, all information about these samples is available to the user, including cell‐level data and memberships of individual cells in each population. The gating tree can be visualized (Fig. 1) as well as the individual dot plots of the gating scheme for each sample. One such sample is shown in Figure 2.

### Combining Manual and Computational Analysis

**Figure 3:**
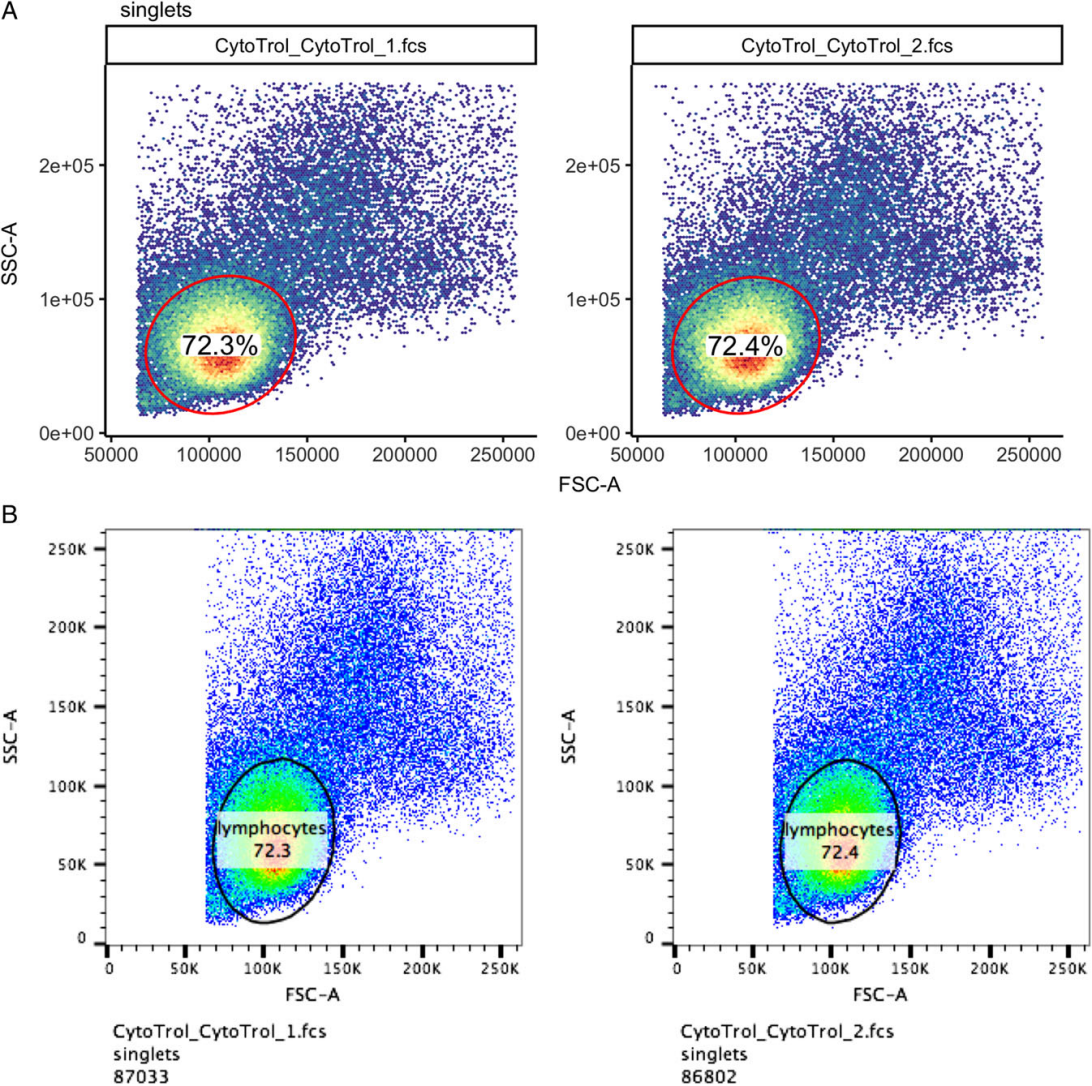
Computational *openCyto* gates of lymphocytes in the forward and side scatter dimensions on two Cytotrol T cell FlowCAP IV samples, (A) visualized using ggcyto in R/Bioconductor, (B) exported with *CytoML* and visualized in FlowJo. [Color figure can be viewed at wileyonlinelibrary.com].

Once data have been imported they can be further analyzed using computational methods. We use the *openCyto* package 23 to gate the lymphocyte population in the forward and side scatter dimensions, modifying the imported *GatingSet* object. The resulting gates, visualized with *ggcyto* are shown in Figure 3A.

High dimensional clustering can also be combined with manual gating by leveraging the cytometry infrastructure available in R. We show an example how *FlowSOM* clustering can be applied to the lymphocyte population defined above and exported to FlowJo (see Supporting Information Material:” Combining manual analysis with high dimensional clustering” and Supporting Information Figs. S3 and S4). The clustering results can be exported by *CytoML*, but this is currently only supported for FlowJo (see Supporting Information Fig. S5). This is due to a lack of Gating‐ML support for high dimensional clustering results in general, requiring ad‐hoc solutions for each platform.

### Exporting Gated Data to FlowJo

The GatingSet2flowJo() API is used to create a FlowJo–compatible workspace from the modified *GatingSet*. The newly created workspace is opened FlowJo and the lymphocyte gates visualized therein (Fig. 3B).

### Exporting Gated Data to Cytobank

**Figure 4:**
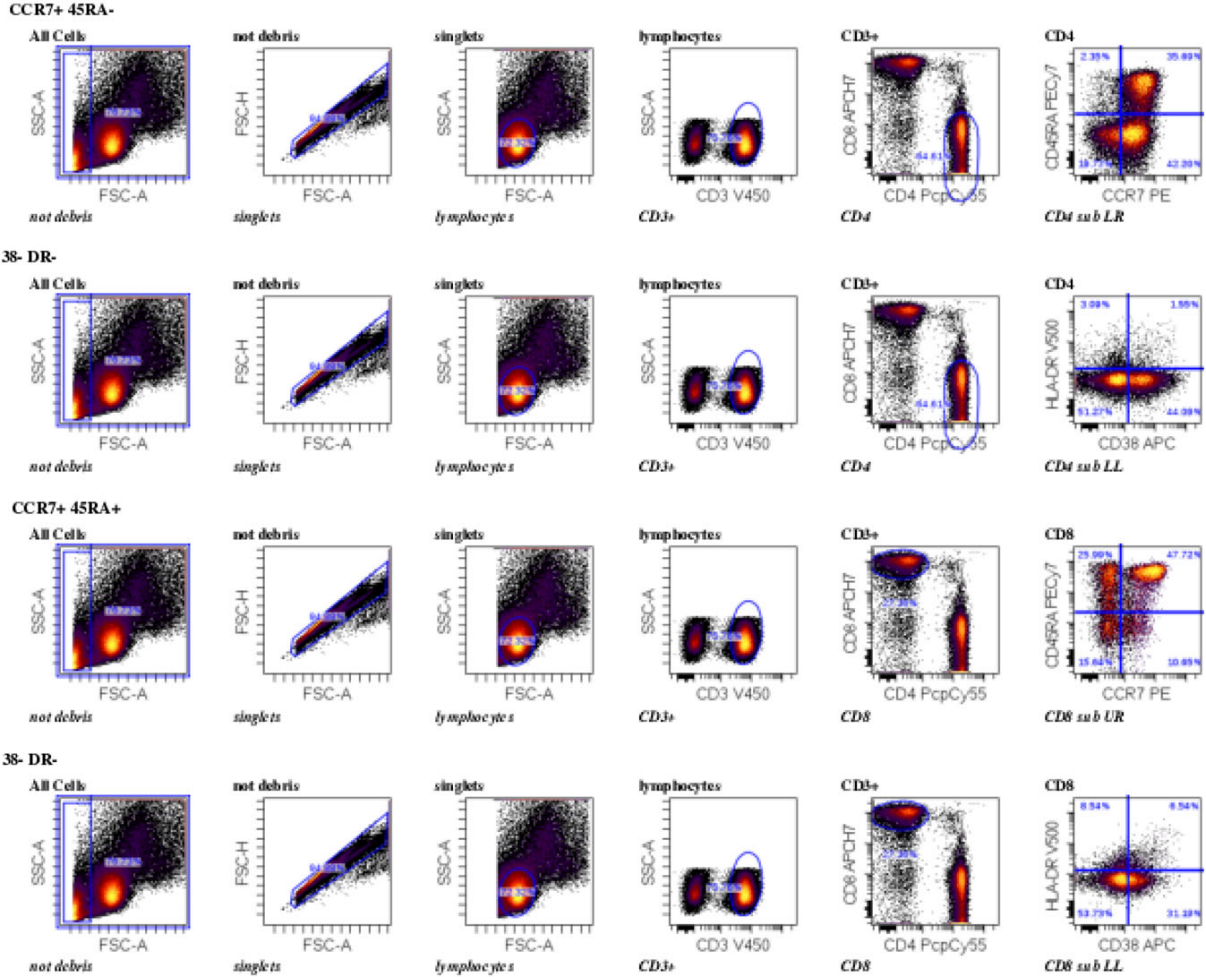
The gating hierarchy for one Cytotrol T cell sample from FlowCAP IV, with openCyto computational gates on lymphocytes, exported using *CytoML* to Cytobank format and visualized in Cytobank. [Color figure can be viewed at wileyonlinelibrary.com].

The GatingSet2cytobank() API is used to export the data to a Cytobank–compatible format. After export, the FCS files, and the workspace containing the gates were imported into Cytobank. These are available under experiment id 138,779 (https://community.cytobank.org/cytobank/experiments/72951/illustrations/138779) The Cytobank visualization of the gating hierarchy is shown in Figure 4, and in the online illustration above.

### Importing Gated Data from Cytobank

Finally, we demonstrate how to import Cytobank data and gates into R to reproduce the gating of a public Cytobank experiment “Kinase‐Inhibitor Treated Reprogramming MEFs.”

**Figure 5:**
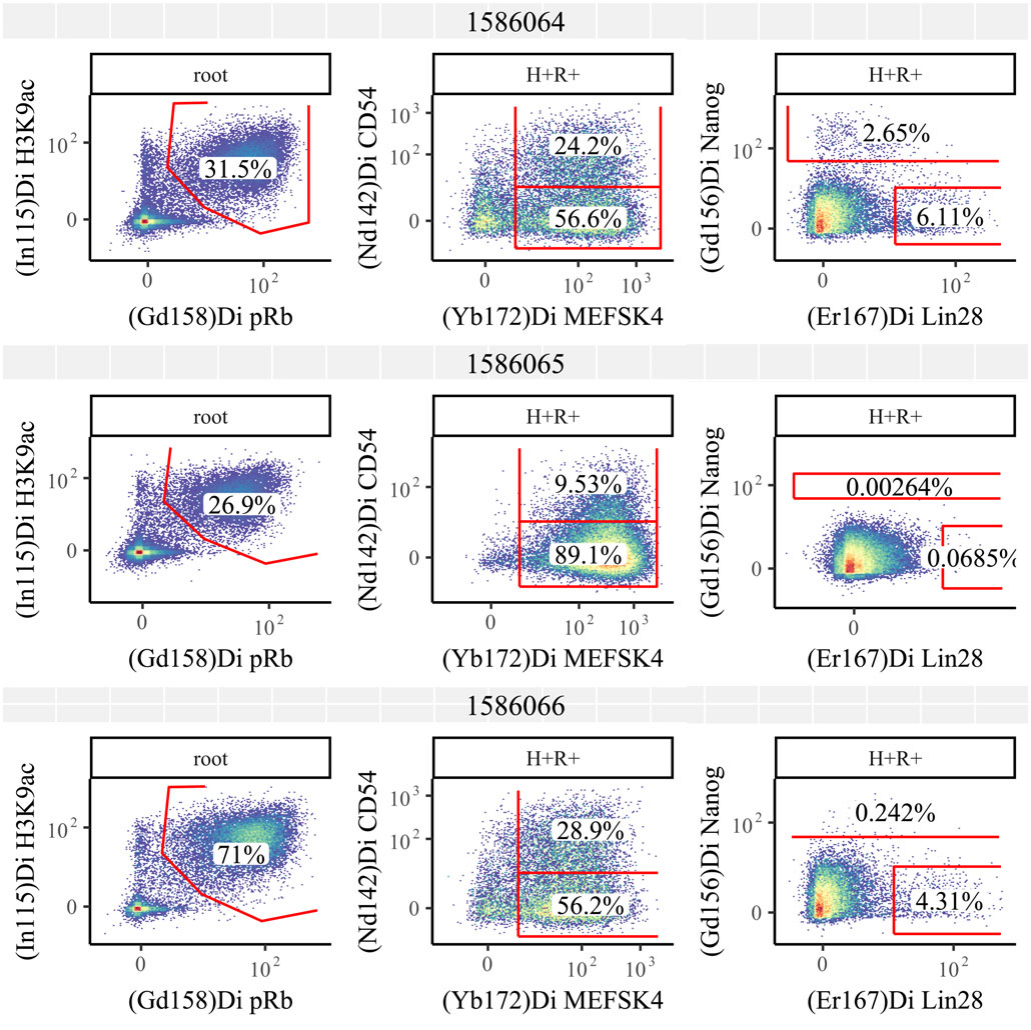
A *ggcyto* visualization of three samples from a public Cytobank experiment (id 43,281) “kinase inhibitor‐treated reprogramming MEFs,” imported into R using *CytoML*. [Color figure can be viewed at wileyonlinelibrary.com].

The ACS container was downloaded, “unzipped” and the contained xml workspace, along with the FCS files imported using the cytobank2GatingSet() API in the *CytoML* package. The gates for three of the six samples in this experiment are shown in Figure 5, visualized using *ggcyto*.

### A Common Underlying Data Representation Enables Reproducibility

The *CytoML* import function transforms cytometry data and gates from FCS and Gating‐ML into Bioconductor objects (*GatingSet* and *GatingHierarchy* implemented in the *flowWorkspace* package). Exporting converts the Bioconductor objects into platform‐specific workspaces containing Gating‐ML representations of gates, data transformations, and compensation matrices. The common representation allows users to convert analyses between platforms in a reproducible manner, incorporating both manual and computational approaches, and allows data to be shared between researchers using those platforms. Other platforms can be supported in the future but will require those platforms to open up their workspace file formats.

## Features and Limitations

One of the principal strengths of *CytoML* is the ability to import and reproduce a data analysis in R, bringing along the gates, data transformations, and compensation matrices, and cell‐level data. This allows users to leverage *flowWorkspace*, *ggcyto* and other R packages to interrogate, manipulate and modify, and visualize the underlying single‐cell data. Although computational and non‐computational analyses using traditional bivariate gates can be combined and exported for visualization on other platforms or within the R framework itself (Supporting Information Material and Figs. 2, 3, 4), access to the cell‐level data also allows users to perform high‐dimensional clustering, which can be readily attached to the R gating representation (Supporting Information Material: “Combining manual analysis with high dimensional clustering”), and can be compared against manual gating (Supporting Information Material: “Accessing cell level data,” Supporting Information Figs. S3 and S4). Lack of standardized support for cluster results in Gating‐ML is limiting, and export of high dimensional clustering by *CytoML* is currently only supported for visualization in FlowJo. Support for other platforms will be incorporated in future releases.

## Conclusion

*CytoML* enables users to import gated cytometry data from multiple commonly used analysis platforms (Diva, FlowJo, and Cytobank) into R for visualization and further analysis. Data can be further analyzed within R using manual or computational approaches and the gates and clustering results exported to FlowJo or Cytobank (cluster export not supported for Cytobank at this time). This allows users of those platforms to visualize and explore results from computational analyses or from other platforms more easily. We envision that *CytoML* will facilitate collaboration and data sharing between computational and non‐computational researchers in cytometry, and importantly will motivate reproducible cytometry analysis by helping users and reviewers validate computational and manual analyses and analysis pipelines.

## Conflicts of Interest

The authors declare that they have no conflicts of interest.

## Supporting information

Supplementary Material
